# The emergency department arrival mode and its relations to ED management and 30-day mortality in acute heart failure: an ancillary analysis from the EURODEM study

**DOI:** 10.1186/s12873-022-00574-z

**Published:** 2022-02-14

**Authors:** Pia Harjola, Tuukka Tarvasmäki, Cinzia Barletta, Richard Body, Jean Capsec, Michael Christ, Luis Garcia-Castrillo, Adela Golea, Mehmet A. Karamercan, Paul-Louis Martin, Òscar Miró, Jukka Tolonen, Oene van Meer, Ari Palomäki, Franck Verschuren, Veli-Pekka Harjola, Said Laribi, On Behalf of the EURODEM Study Group, Patrick Plaisance, Ghanima Al Dandachi, Maxime Maignan, Dominique Pateron, Christelle Hermand, Cindy Tessier, Pierre-Marie Roy, Lucie Bucco, Nicolas Duytsche, Pablo Garmilla, Giorgio Carbone, Roberto Cosentini, Sorana Truță, Natalia Hrihorișan, Diana Cimpoeșu, Luciana Rotaru, Alina Petrică, Mariana Cojocaru, Silvia Nica, Rodica Tudoran, Cristina Vecerdi, Monica Puticiu, Titus Schönberger, Constant Coolsma, Maarten Baggelaar, Noortje Fransen, Crispijn van den Brand, Doutsje Idzenga, Maaike Maas, Myriam Franssen, Charlotte Mackaij Staal, Lot Schutte, Marije de Kubber, Lisette Mignot-Evers, Ursula Penninga-Puister, Joyce Jansen, Jeroen Kuijten, Marna Bouwhuis, Adam Reuben, Jason Smith, Shammi Ramlakhan, Melanie Darwent, James Gagg, Liza Keating, Santosh Bongale, Elaine Hardy, Jeff Keep, Heather Jarman, Steven Crane, Olakunle Lawal, Taj Hassan, Alasdair Corfield, Matthew Reed, Felicitas Geier, Yvonne Smolarsky, Sabine Blaschke, Clemens Kill, Andreas Jerrentrup, Christian Hohenstein, Felix Rockmann, Tanja Brünnler, Alexandre Ghuysen, Marc Vranckx, Mehmet A. Karamercan, Mehmet Ergin, Zerrin D. Dundar, Yusuf A. Altuncu, Ibrahim Arziman, Mucahit Avcil, Yavuz Katirci, Hanna Suurmunne, Liisa Kokkonen, Juha Valli, Minna Kiljunen, Jukka Tolonen, Sanna Kaye, Mikko Mäkelä, Juhani Metsäniitty, Eija Vaula

**Affiliations:** 1 Emergency Medicine, University of Helsinki and Department of Emergency Medicine and Services, Helsinki University Hospital, Helsinki, Finland; 2 Cardiology, University of Helsinki and Heart and Lung Cent, Helsinki University Hospital, Helsinki, Finland; 3 Servicio Urgencias Hospital Marqués de Valdecilla, Santander, Spain; 4 Division of Cardiovascular Sciences, The University of Manchester, Manchester, UK; 5 Emergency Department, Central Manchester University Hospitals NHS Foundation Trust, Oxford Road, Manchester, England; 6 Department of Public Health, Centre Hospitalier Régional Universitaire de Tours, Tours, France; 7 Department of Emergency Care, Luzerner Kantonsspital, Luzern, Switzerland; 8 Emergency Medicine, County Emergency Hospital Cluj-Napoca, University of Medicine and Pharmacy, Cluj-Napoca, Romania; 9 Emergency Medicine Department, Faculty of Medicine, Gazi University, Ankara, Turkey; 10 Department of Emergency Medicine, Istanbul Bagcilar Training and Research Hospital, Istanbul, Turkey; 11 School of Medicine and CHU Tours, Emergency Medicine Department, Tours University, Tours, France; 12 Emergency Department, Hospital Clínic, University of Barcelona, Barcelona, Catalonia Spain; 13 Internal Medicine, University of Helsinki and Department of Medicine, Helsinki University Hospital, Helsinki, Finland; 14 Leiden University Medical Center, Leiden, The Netherlands; 15 Emergency Medicine, Campus of Tampere, Kanta-Häme Central Hospital, Hämeenlinna, Finland; 16 Department of Acute Medicine, Université Catholique de Louvain, Cliniques Universitaires Saint-Luc, Brussels, Belgium

**Keywords:** Acute heart failure, Arrival mode, Management, Prognosis, Emergency medical services, Ventilatory support

## Abstract

**Background:**

Acute heart failure patients are often encountered in emergency departments (ED) from 11% to 57% using emergency medical services (EMS). Our aim was to evaluate the association of EMS use with acute heart failure patients’ ED management and short-term outcomes.

**Methods:**

This was a sub-analysis of a European EURODEM study. Data on patients presenting with dyspnoea were collected prospectively from European EDs. Patients with ED diagnosis of acute heart failure were categorized into two groups: those using EMS and those self-presenting (non- EMS). The independent association between EMS use and 30-day mortality was evaluated with logistic regression.

**Results:**

Of the 500 acute heart failure patients, with information about the arrival mode to the ED, 309 (61.8%) arrived by EMS. These patients were older (median age 80 vs. 75 years, *p* < 0.001), more often female (56.4% vs. 42.1%, *p* = 0.002) and had more dementia (18.7% vs. 7.2%, *p* < 0.001). On admission, EMS patients had more often confusion (14.2% vs. 2.1%, *p* < 0.001) and higher respiratory rate (24/min vs. 21/min, *p* = 0.014; respiratory rate > 30/min in 17.1% patients vs. 7.5%, *p* = 0.005). The only difference in ED management appeared in the use of ventilatory support: 78.3% of EMS patients vs. 67.5% of non- EMS patients received supplementary oxygen (*p* = 0.007), and non-invasive ventilation was administered to 12.5% of EMS patients vs. 4.2% non- EMS patients (*p* = 0.002). EMS patients were more often hospitalized (82.4% vs. 65.9%, *p* < 0.001), had higher in-hospital mortality (8.7% vs. 3.1%, *p* = 0.014) and 30-day mortality (14.3% vs. 4.9%, *p* < 0.001). The use of EMS was an independent predictor of 30-day mortality (OR = 2.54, 95% CI 1.11–5.81, *p* = 0.027).

**Conclusion:**

Most acute heart failure patients arrive at ED by EMS. These patients suffer from more severe respiratory distress and receive more often ventilatory support. EMS use is an independent predictor of 30-day mortality.

## Background

Acute heart failure (AHF) is a complex, heterogenous and often life-threatening clinical syndrome. It is a frequent cause for hospitalization and constitutes a significant proportion of patients, especially with dyspnoea, transferred by emergency medical services (EMS) to the emergency departments (ED) [1–3]. The proportion of AHF patients arriving at the ED by EMS varies from 11% to 57% [4–10]. Overall, the prognosis of AHF remains poor; in-hospital mortality ranging from 3.8% to 6.6% [11–14] and on average one fifth of AHF patients dying during one year follow up [12, 15]. Patients using EMS are reported to have higher in-hospital and 30-day mortality compared to those self-presenting to the ED [7, 8]. 

The main complaint of AHF is shortness of breath [6, 13]. One of the main goals of AHF management (in addition to stabilisation of hemodynamic) is to relieve patients’ symptoms and to reduce fluid overload. Intravenous (IV) diuretics and vasodilators are the mainstay of AHF management [16]. Registries show that approximately 80% of AHF patients are treated with IV diuretics [4, 14, 17–21]. However, less than half of AHF patients receive IV vasodilators [4, 14, 17–20, 22], and non-invasive ventilation (NIV) is administered to 7–20% of AHF patients [4, 5, 14, 18, 20, 22, 23]. Data on the association between EMS arrival and the ED management of AHF are, however, lacking.

Earlier studies regarding AHF patients’ EMS use have focused mainly on clinical factors associated with the use and the prognostic effects of EMS [7, 8]. The aim of this study was to determine whether the arrival mode is associated with the AHF management in ED, in addition to the patient outcomes.

## Methods

This study was a sub-analysis of the prospective, multinational EURODEM study [24]. The EURODEM study included patients presenting to ED with shortness of breath, dyspnoea being one of the symptoms listed in the triage on ED admission. The data was collected in three 72-h periods (February, May and October 2014) by local ED nurses or physicians. ED diagnoses were recorded. The physician made the ED diagnosis based on patient history, clinical assessment, imaging, and laboratory tests. Patients with ED diagnosis of AHF were included to this analysis. The AHF patients were categorized based on their ED arrival mode: those arriving by EMS (EMS patients) and those self-presenting (non-EMS patients). The collected data included patient characteristics, initial assessment (clinical assessment and vital signs), laboratory tests, ED management, in-hospital outcomes, and 30-day mortality. The 30-day outcome was ascertained by a follow-up phone call. The study was performed in accordance with the Declaration of Helsinki. The approval of local ethics committee was received from all participating centres according to local requirements. In most participating centres patient consent for data collection was received.

Respiratory distress was defined as respiratory rate (RR) > 30 breaths/min. The peripheral oxygen saturation (SpO_2_) was measured after 30-min oxygenation. Categorical variables are reported as numbers and percentages (%) and continuous variables as medians with interquartile range (IQR). Between-group comparisons were performed with chi-square test for categorical variables and Mann–Whitney U test for continuous variables. Independent predictors associated with 30-day mortality were analysed with multivariable logistic regression. To reduce bias and to maximise sample size, variables with missing data 20% at most were included using the multiple imputation method with 20 imputations. The initial selection of variables was based on clinical relevance and previous literature [25–28]. Forward and backward logistic regression was used for the final variable selection from the following variables: age, gender, ED arrival mode (i.e., EMS), systolic blood pressureSBP, heart rate (HR), RR, SpO_2_, sodium, potassium, haemoglobin, confusion, a history of chronic obstructive pulmonary disease (COPD), active cancer, chronic kidney disease (CKD), and cognitive dysfunction/dementia. P-value significance < 0.05 was used for inclusion and > 0.1 for elimination.

IBM SPSS version 25 was used for statistical analysis. A p-value below 0.05 was considered statistically significant.

## Results

The EURODEM study included 2525 patients of which 507 had AHF as ED diagnosis. Data from the arrival mode was missing from seven patients, which were excluded from the analyses. The majority of AHF patients (*n* = 309 (61.8%)) arrived at the ED by EMS. Compared to non-EMS patients, EMS patients were older and more often female (Table 1).

**Table 1:** Patient characteristics

	**All, *n* = 500**		**EMS, *n* = 309**		**Non-EMS, *n* = 191**		***P*-value**
		missing (n)		missing (n)		missing (n)	
**Demographics**							
Age, years	78 (69–84)		80 (71–85)		75 (65–81)		< 0.001
Duration of symptoms (days)	3 (1–7)		3 (1–7)		3 (2–10)		0.002
Gender (male), *n* (%)	244 (49.1)		134 (43.6)		110 (57.9)		0.002
**Comorbidities, *n* (%)**							
Previous heart failure	290 (60.9)		180 (61.6)		110 (59.8)		0.685
Diabetes	188 (38.2)		100 (32.8)		88 (47.1)		0.002
Hypertension	348 (71.0)		225 (73.8)		123 (66.5)		0.085
Prior atrial fibrillation	175 (35.7)		110 (36.3)		65 (34.8)		0.729
COPD	128 (27.6)		82 (28.8)		46 (25.8)		0.493
Smoker	79 (17.9)	61 (12.2)	49 (18.4)	43 (13.9)	30 (17.0)	15 (7.9)	0.712
Asthma	32 (6.8)		19 (6.5)		13 (7.3)		0.725
Ischemic heart disease	197 (41.6)		120 (41.1)		77 (42.5)		0.757
Dyslipidaemia	168 (35.8)		93 (32.5)		75 (41.0)		0.062
Chronic kidney disease	119 (25.2)		80 (27.4)		39 (21.7)		0.164
Valvular disease	86 (18.1)		57 (19.5)		29 (15.9)		0.333
Anaemia	76 (16.3)		47 (16.4)		29 (16.2)		0.947
Active cancer	30 (6.5)		17 (6.0)		13 (7.3)		0.572
Prior PE	21 (4.3)		19 (6.3)		2 (1.1)		0.007
Obesity	117 (24.9)		75 (25.8)		42 (23.5)		0.574
Dementia	67 (14.3)		54 (18.7)		13 (7.2)		0.001

The values are given either as number (%) or median (interquartile range)

*COPD* Chronic obstructive pulmonary embolism

*PE* Pulmonary embolism

A total of 290 (60.9%) patient had a previous diagnosis of HF, but no significant differences existed between the groups. EMS patients had significantly more often dementia and a history of pulmonary embolism, whereas diabetes was more common in non-EMS patients. No other major differences in the prevalence of comorbidities were observed between the groups (Table 1). The median duration of dyspnoea before ED admission was 3 days in both groups (EMS 3 (1–7) days vs non-EMS 3 (2–10) days, *p* = 0.002) (Table 1).

On admission to ED, the median SBP of all AHF patients was 140 (120–159) mmHg and HR 88 (75–110) beats per minute. No significant differences appeared in BP and HR levels between the groups (Table 2). EMS patients had significantly higher RR compared to non-EMS patients. The median SpO_2_ after 30 min oxygenation was 94% in both groups. Most AHF patients had rales on lung auscultation. EMS patients had significantly more often abnormal breath sounds (Table 2).

**Table 2:** Clinical characteristics on admission to emergency department

	**All AHF, *n* = 500**		**EMS- patients, *n* = 309**		**Non-EMS- patients, *n* = 191**		***P*-value**
**Vital signs**		Missing (n)		Missing(n)		Missing (n)	
SBP < 100 mmHg, *n* (%)	27 (5.5)		18 (5.9)		9 (4.8)		0.597
SBP > 140 mmHg, *n* (%)	243 (49.3)		146 (47.9)		97 (51.6)		0.421
SBP (mmHg)	140 (120–159)		140 (120–156)		143 (122–162)		0.285
DBP (mmHg)	80 (66–91)		80 (66–92)		80 (67–90)		0.801
Heart rate (bpm)	88 (75–110)		90 (75–110)		85 (75–104)		0.115
Heart rate > 100 bpm, *n* (%)	160 (32.5)		111 (36.4)		49 (26.2)		0.019
Hear rate > 120 bpm, *n* (%)	52 (10.6)		38 (12.5)		14 (7.5)		0.082
Respiratory rate, (per min)	22 (18–28)		24 (19–30)		21 (18–26)		0.014
Respiratory rate > 30/min, n (%)	55 (13.3)	89 (17.8)	43 (17.1)	58 (18.8)	12 (7.5)	30 (15.7)	0.005
SpO_2_ (%) with supplementary O_2_	94 (90–97)		94 (90–97)		94 (89–96)		0.569
SpO_2_ < 90% with supplementary O_2_, *n* (%)	118 (24.5)		71 (23.7)		47 (25.8)		0.607
Temperature (°C)	36.5 (36.0–36.9)		36.5 (36.0–37.0)		36.5 (36.0–36.8)		0.262
**Clinical signs, *n* (%)**							
Rales on auscultation	346 (71.8)		228 (76.8)		118 (63.8)		0.002
Wheezing on auscultation	87 (19.7)	58 (11.6)	62 (23.1)	41 (13.2)	25 (14.4)	17 (8.9)	0.024
Peripheral oedema	273 (56.2)		173 (58.1)		100 (53.2)		0.293
Jugular vein distension	110 (25.1)	62 (12.4)	72 (27.4)	46 (14.9)	38 (21.7)	16 (8.4)	0.181
Confusion	47 (9.6)		43 (14.2)		4 (2.1)		< 0.001
**Laboratory parameters**							
NT-proBNP (pg/mL)	3661 (1328–10,377)	381 (76.2)	5144 (1846–11205)	223 (72.2)	2103 (688–5167)	155 (81.2)	0.001
Creatinine (μmol/L)	101 (78–136)		107 (77–137)		94 (80–131)		0.751
Sodium (mmol/L)	138 (136–141)		138 (135–141)		138 (136–141)		0.476
Potassium (mmol/L)	4.3 (4.0–4.8)		4.3 (4.0–4.8)		4.3 (3.9–4.7)		0.243
CRP (mg/dL)	10 (4–30)		13 (5–41)		8 (3–20)		0.003
pH	7.40 (7.34–7.45)		7.38 (7.32–7.44)		7.43 (7.37–7.46)		0.001
PaCO_2_ (mmHg)	38.0 (31.6–45.5)		39.2 (31.5–46.0)		36.0 (32.0–43.1)		0.296
White cell count (10^9^/L)	9.0 (7.0–11.5)		9.0 (7.0–13.0)		8.8 (6.8–10.2)		0.070
Haemoglobin (g/dL)	12.3 (10.7–13.9)		12.2 (10.6–13.7)		12.3 (10.7–14.0)		0.626
Haemoglobin < 100 g/L, *n* (%)	58 (12.9)	51 (10.2)	38 (13.8)	33 (10.7)	20 (11.6)	18 (9.4)	0.497

The values are given either as number (%) or median (interquartile range)

*SBP* Systolic blood pressure

*DBP* Diastolic blood pressure

*SpO*_*2*_ Peripheral oxygen saturation

**Fig. 1:**
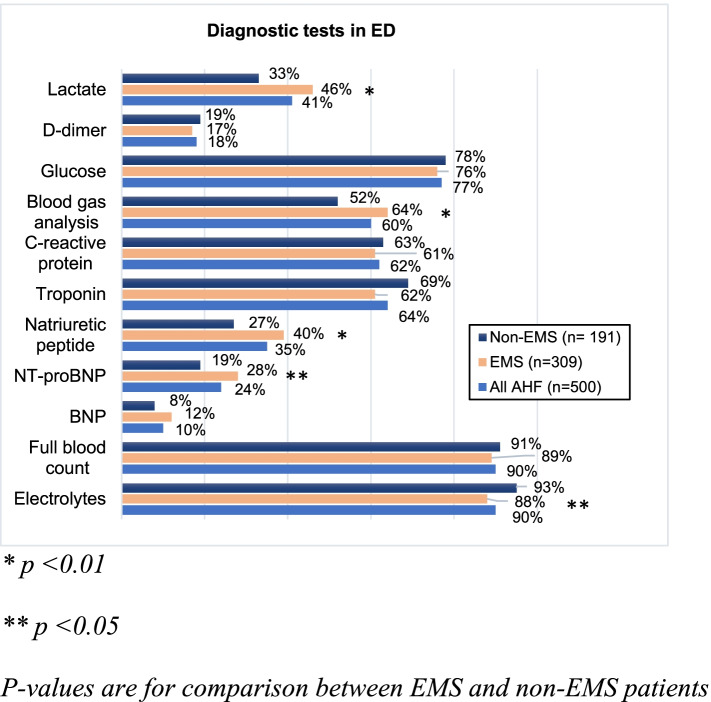
The frequency of diagnostic tests on admission to emergency department, ED = Emergency department

Regarding laboratory tests, NT-proBNP was measured in 24.4% of AHF patients and BNP in 10.4% of patients. NT-proBNP was measured significantly more often in EMS patients (Fig. 1) and the levels were significantly higher (Table 2). The median pH of all AHF patients was 7.40 (7.34–7.45). EMS patients had lower blood pH values (Table 2).

**Fig. 2:**
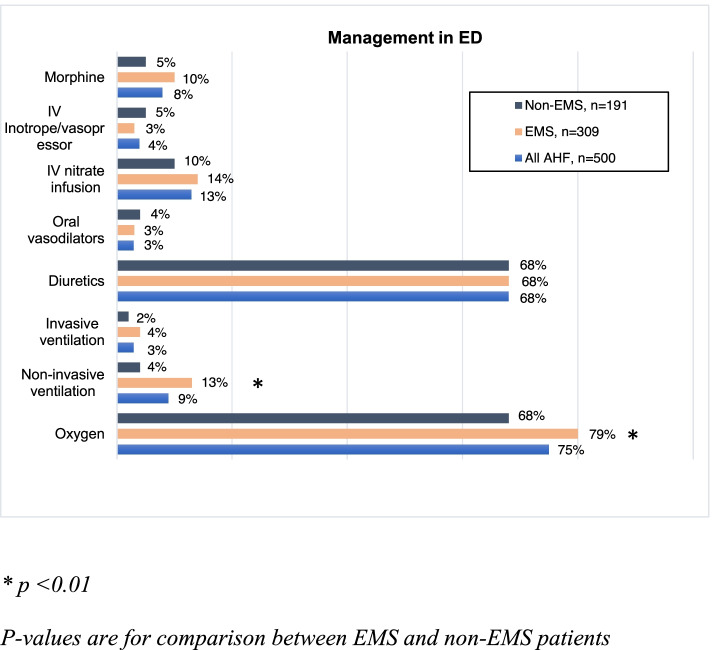
Acute heart failure management on admission to emergency department, ED = Emergency department

Figure 2 shows the frequency of AHF management in the ED. IV diuretics were administered to 68% of AHF patients, whereas nitrate infusion to 13%. The only significant difference in the use of ED management between the two groups appeared in ventilatory support, which was significantly more often provided to EMS patients: supplementary oxygen to 78.3% EMS vs. 67.5% non-EMS patients (*p* = 0.007), and NIV to 12.5% EMS vs. 4.2% non-EMS patients (*p* = 0.002). In univariate analysis lower SpO_2_ (*p* < 0.001) and higher RR (*p* < 0.001) were associated with NIV use. Three percentage of patients received mechanical ventilation; no difference appeared between the patient groups. Patients with confusion were intubated significantly more often compared to the rest of the AHF patients (21.7% vs. 0.9%), *p* < 0.001).

**Fig. 3:**
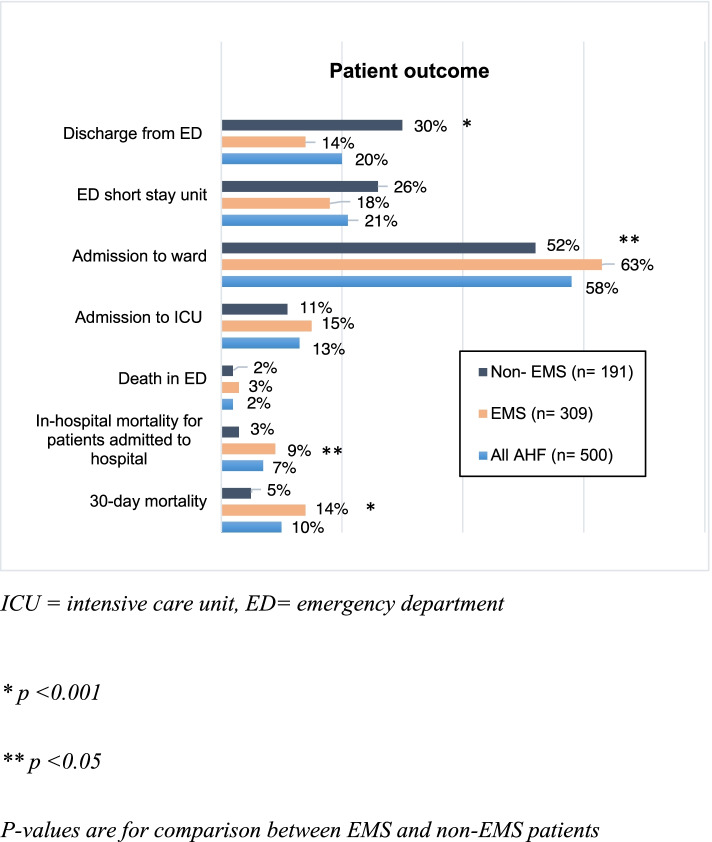
Patient outcome

Patient outcomes are shown in Fig. 3. Three fourths of all AHF patients were hospitalized from the ED, EMS patients more frequently compared to non-EMS. More than half of all AHF patients were admitted to a ward, EMS patients more often. The median length of hospital stays (LOS) was 7 (2–12) days in both groups. The all-cause in-hospital mortality was 6.6% (8.7% EMS patients vs. 3.1% non-EMS patients, *p* = 0.014). The 30-day mortality was significantly higher in the EMS group (14.3% vs. 4.9% non-EMS patients, *p* < 0.001).

Variables included in the final multivariable logistic regression model in 30-day mortality prediction included gender, EMS, SpO_2,_ sodium, haemoglobin, and confusion; the final model was further adjusted with age. EMS was an independent predictor of 30-day mortality (OR = 2.54, 95% CI 1.11–5.81, *p* = 0.027). The other independent predictors were male gender (OR = 2.75, 95% CI 1.32–5.76, *p* = 0.007), confusion (OR = 5.28, 95% CI 2.30 -12.16, *p* < 0.001), SpO_2_ (OR = 0.94, 95% CI 0.90–0.98, *p* = 0.008), sodium level (OR = 0.91, 95% CI 0.86–0.96, *p* = 0.002), and haemoglobin (OR = 0.80, 95% CI 0.69–0.92, *p* = 0.002).

## Discussion

This sub-analysis describes the association of ED arrival mode with AHF management in the ED and patient outcomes in European centres. First, this study shows that the majority of AHF patients arrived at the ED by EMS. These patients were more likely to be older females with more dementia compared to the patients self-presenting. Second, patients arriving at the ED by EMS suffered more often from respiratory distress, and consequently received more often ventilatory support. However, no other differences were observed in the administration frequencies of AHF treatments in the ED. Third, EMS patients had worse short-term outcomes and the use of EMS was an independent predictor of 30-day mortality.

In the present study more than half of AHF patients arrived at the ED by EMS; the proportion is among the highest in the literature [4–10, 29]. In line with the previous studies, older women were more prone to use EMS [6–8]. However, the comorbidities associated with the EMS use differ between these studies. In line with a previous study, EMS patients had more often dementia [6–8]. While other cardiovascular diseases, COPD and CKD have been more common among EMS patients in the other studies, we found only a history of pulmonary embolism to be more frequent among EMS patients compared to non-EMS patients. In addition, two thirds of the patients had a history of HF but contrary to some of the earlier studies no significant difference occurred between EMS and non-EMS patients [6, 7].

Although most initial parameters did not differ between the patient groups, EMS patients suffered more often from respiratory distress and confusion, as well as more severe cardiac stress, reflecting more severe clinical presentation of AHF, especially pulmonary oedema [30]. As also reported earlier [8], the EMS patients had shorter duration of symptoms before ED admission, perhaps reflecting the more abrupt and severe clinical presentation. The association of severity of illness and EMS referral has also been observed previously [7].

Presumably the most measured laboratory parameters were full blood count and electrolytes as the availability is high. In contrast the use of natriuretic peptides was the lowest. Yet, the natriuretic peptids are recommended to be measured mainly to rule out AHF and when diagnosis of AHF is uncertain, not in all AHF patients [31]. In addition, the use of lactate measurement was among the lowest but in light of the esc guidelines, which recommend measuring  it from patients suspected cardiogenic shock [31], at least adequate when considering the number of hypotensive patients in the study.

Respiratory distress is one of the most common reasons for EMS use [1], as confirmed by our study. Due to more severe respiratory distress, EMS patients received more often ventilatory support compared to their counterparts. Naturally, the use of NIV and supplementary oxygen were associated to higher RR and lower SpO2. However, even though the use of NIV in the EMS group was similar to earlier studies [4, 5, 14, 18, 20, 22, 23], it might have been indicated more often with regard to the ESC guidelines [16]—as one fourth of all the AHF patients had SpO_2_ less than 90% with supplementary oxygen. On the other hand, confusion—one of the contraindications for NIV use—could partly explain the relatively low frequency of NIV use. Moreover, one fifth of all the patients reported to have confusion were intubated, which was significantly more often compared to the rest of the AHF patients.

Since the clear majority of AHF patients present with congestion without hypoperfusion and hypotension [16, 17, 32], vasodilators and diuretics are the mainstay of AHF management [16]. Although only a minority of all AHF patients in our study were hypotensive and half hypertensive, only one patient out of eight received nitrate infusion. The underuse of vasodilators has been observed previously as well [17, 18, 21]. In contrast, diuretics were given to more than two thirds of all patients. All in all, there is room for improvement in the ED management of AHF as also pointed out earlier [7].

Finally, EMS patients were more often admitted to a ward and, in line with previous studies, had significantly higher in-hospital [7, 10] and 30-day mortality [7, 8, 10, 33]. Indeed, EMS patients were older, had more often dementia and were more severely ill and the same factors related to EMS use – lower SpO_2_ and confusion– were also observed among the independent predictors of 30-day mortality. Notably, confusion was a strong marker for increased risk of death as well, which warrants assessing mental state not only in the most severe AHF, i.e. cardiogenic shock [34], but in all AHF patients. Still, as also shown earlier [8, 10, 33], EMS use remained an independent predictor of 30-day mortality, which is likely linked to patients’ fragility and illness severity affected by unmeasured and unknown confounders, as well to patient preference.

This study corroborates the perception that AHF patients transported by EMS are, on average, older women with dementia suffering more often from dyspnoea and respiratory distress. All in all, the use of EMS in Europe seems appropriate. However, the more severe clinical presentation, worse outcomes, and possible underuse of AHF treatments necessitate the need for increased vigilance in identifying these patients, especially from other dyspnoeic patients, and treating them appropriately.

## Limitations

Some limitations need to be addressed. First, this was an observational study and the association between EMS and 30-day mortality must be interpreted with caution due to possible unknown and unmeasured confounders. Second, as this is a sub-analysis of a large multi-centre study, the number of AHF patients was rather small in some countries and participating centres, and no random effects model considering the country/centre was performed. Third, we didn’t have information about the criteria for EMS referral in different EMS regions. Fourth, the doses of AHF medications were not registered which might have differed between EMS and non-EMS patients due to difference in disease severity even though the overall use of AHF medications was similar. Fifth, there was a fair amount of data missing not at random in some of in some of the biochemistry variables, and thus not used in the multiple imputation and the regression analyses.

## Conclusion

In conclusion, our study shows that the majority of AHF patients arrive at the ED by EMS. Older age, female gender, dementia, confusion, and especially respiratory distress seem to be the driving forces for EMS use. Apart from the more frequent use of ventilatory support, the use of AHF treatments in the ED does not differ between EMS and non-EMS patients. EMS patients are more often admitted to a ward and the use of EMS is an independent predictor of 30-day mortality. More prospective research should be done in the pre-hospital phase to discover the reasons for differences in the outcomes between EMS and non-EMS patients.

## Data Availability

The EURODEM database is not publicly available. The datasets used and/or analyzed during the current study are available from the corresponding author on reasonable request.

## Abbreviations

AHF: Acute heart failure
ED: Emergency department
EMS: Emergency medical services
IV: Intravenous
NIV: Non-invasive ventilation
SBP: Systolic blood pressure
RR: Respiratory rate
SpO_2_: Peripherial oxygen saturation
IQR: Inter quartile range
HR: Heart rate
COPD: Chronic obstructive pulmonary disease
LOS: Length of hospital stay
CKD: Chronic kidney disease

## References

[1] Prekker ME, Feemster LC, Hough CL (2014). The epidemiology and outcome of prehospital respiratory distress. Acad Emerg Med.

[2] Kelly AM, Holdgate A, Keijzers G (2016). Epidemiology, prehospital care and outcomes of patients arriving by ambulance with dyspnoea: an observational study. Scand J Trauma Resusc Emerg Med.

[3] Kauppi W, Herlitz J, Magnusson C (2020). Characteristics and outcomes of patients with dyspnoea as the main symptom, assessed by prehospital emergency nurses- a retrospective observational study. BMC Emerg Med.

[4] Chouihed T, Manzo-Silberman S, Peschanski N (2016). Management of suspected acute heart failure dyspnea in the emergency department: results from the French prospective multicenter DeFSSICA survey. Scand J Trauma Resusc Emerg Med.

[5] Logeart D, Isnard R, Resche-Rigon M (2013). Current aspects of the spectrum of acute heart failure syndromes in a real-life setting: the OFICA study. Eur J Heart Fail.

[6] Harjola P, Boyd J, Tarvasmaki T (2017). The impact of emergency medical services in acute heart failure. Int J Cardiol.

[7] Miro O, Llorens P, Escalada X (2017). Prehospital emergency care of patients with acute heart failure in Spain: the SEMICA study (Emergency Medical Response Systems for Patients with Acute Heart Failure). Emergencias.

[8] Ezekowitz JA, Podder M, Hernandez AF (2016). Arrival by ambulance in acute heart failure: insights into the mode of presentation from Acute Studies of Nesiritide in Decompensated Heart Failure (ASCEND-HF). BMJ Open.

[9] Llorens P, Javaloyes P, Martin-Sanchez FJ (2018). Time trends in characteristics, clinical course, and outcomes of 13,791 patients with acute heart failure. Clin Res Cardiol..

[10] Wong YW, Fonarow GC, Mi X (2013). Early intravenous heart failure therapy and outcomes among older patients hospitalized for acute decompensated heart failure: findings from the Acute Decompensated Heart Failure Registry Emergency Module (ADHERE-EM). Am Heart J..

[11] Abraham WT, Fonarow GC, Albert NM (2008). Predictors of in-hospital mortality in patients hospitalized for heart failure: insights from the Organized Program to Initiate Lifesaving Treatment in Hospitalized Patients with Heart Failure (OPTIMIZE-HF). J Am Coll Cardiol.

[12] Chioncel O, Mebazaa A, Harjola VP (2017). Clinical phenotypes and outcome of patients hospitalized for acute heart failure: the ESC Heart Failure Long-Term Registry. Eur J Heart Fail.

[13] Adams KF, Fonarow GC, Emerman CL (2005). Characteristics and outcomes of patients hospitalized for heart failure in the United States: rationale, design, and preliminary observations from the first 100,000 cases in the Acute Decompensated Heart Failure National Registry (ADHERE). Am Heart J.

[14] Nieminen MS, Brutsaert D, Dickstein K (2006). EuroHeart Failure Survey II (EHFS II): a survey on hospitalized acute heart failure patients: description of population. Eur Heart J.

[15] Maggioni AP, Dahlstrom U, Filippatos G (2013). EURObservational Research Programme: regional differences and 1-year follow-up results of the Heart Failure Pilot Survey (ESC-HF Pilot). Eur J Heart Fail.

[16] Ponikowski P, Voors AA, Anker SD (2016). Eur Heart J..

[17] Chioncel O, Mebazaa A, Harjola VP (2017). Clinical phenotypes and outcome of patients hospitalized for acute heart failure: the ESC Heart Failure Long-Term Registry. Eur J Heart Fail.

[18] Tarvasmaki T, Harjola VP, Tolonen J (2013). Management of acute heart failure and the effect of systolic blood pressure on the use of intravenous therapies. Eur Heart J Acute Cardiovasc Care.

[19] Maggioni AP, Anker SD, Dahlstrom U (2013). Are hospitalized or ambulatory patients with heart failure treated in accordance with European Society of Cardiology guidelines? Evidence from 12,440 patients of the ESC Heart Failure Long-Term Registry. Eur J Heart Fail.

[20] Follath F, Yilmaz MB, Delgado JF (2011). Clinical presentation, management and outcomes in the Acute Heart Failure Global Survey of Standard Treatment (ALARM-HF). Intensive Care Med.

[21] Maggioni AP, Dahlstrom U, Filippatos G (2010). EURObservational Research Programme: the Heart Failure Pilot Survey (ESC-HF Pilot). Eur J Heart Fail.

[22] Miro O, Hazlitt M, Escalada X (2018). Effects of the intensity of prehospital treatment on short-term outcomes in patients with acute heart failure: the SEMICA-2 study. Clin Res Cardiol.

[23] Pivetta E, Goffi A, Lupia E (2015). Lung ultrasound-implemented diagnosis of acute decompensated heart failure in the ed: A SIMEU Multicenter Study. Chest.

[24] Laribi S, Keijzers G, van Meer O (2019). Epidemiology of patients presenting with dyspnea to emergency departments in Europe and the Asia-Pacific region. Eur J Emerg Med.

[25] Gil V, Miro O, Schull MJ (2018). Emergency Heart Failure Mortality Risk Grade score performance for 7-day mortality prediction in patients with heart failure attended at the emergency department: validation in a Spanish cohort. Eur J Emerg Med.

[26] Peterson PN, Rumsfeld JS, Liang L (2010). A validated risk score for in-hospital mortality in patients with heart failure from the American Heart Association get with the guidelines program. Circ Cardiovasc Qual Outcomes.

[27] Siirila-Waris K, Lassus J, Melin J (2006). Characteristics, outcomes, and predictors of 1-year mortality in patients hospitalized for acute heart failure. Eur Heart J.

[28] Harjola VP, Lassus J, Sionis A (2015). Clinical picture and risk prediction of short-term mortality in cardiogenic shock. Eur J Heart Fail.

[29] Keijzers G, Kelly AM, Cullen L (2017). Heart failure in patients presenting with dyspnoea to the emergency department in the Asia Pacific region: an observational study. BMJ Open.

[30] Dickstein K, Cohen-Solal A, Filippatos G (2008). ESC guidelines for the diagnosis and treatment of acute and chronic heart failure 2008: the Task Force for the diagnosis and treatment of acute and chronic heart failure 2008 of the European Society of Cardiology. Developed in collaboration with the Heart Failure Association of the ESC (HFA) and endorsed by the European Society of Intensive Care Medicine (ESICM). Eur J Heart Fail.

[31] McDonagh TA, Metra M, Adamo M (2021). 2021 ESC Guidelines for the diagnosis and treatment of acute and chronic heart failure. Eur Heart J.

[32] Javaloyes P, Miro O, Gil V (2019). Clinical phenotypes of acute heart failure based on signs and symptoms of perfusion and congestion at emergency department presentation and their relationship with patient management and outcomes. Eur J Heart Fail.

[33] Lee DS, Schull MJ, Alter DA (2010). Early deaths in patients with heart failure discharged from the emergency department: a population-based analysis. Circ Heart Fail.

[34] Kataja A, Tarvasmaki T, Lassus J (2018). Altered mental status predicts mortality in cardiogenic shock - results from the CardShock study. Eur Heart J Acute Cardiovasc Care.

